# Otomycosis; clinical features, predisposing factors and treatment implications

**DOI:** 10.12669/pjms.303.4106

**Published:** 2014

**Authors:** Khurshid Anwar, Muhammad Shahid Gohar

**Affiliations:** 1 Lt Col Khurshid Anwar, MBBS, FCPS (ENT), Combined Military Hospital, Attock Cantt, Punjab, Pakistan.; 2 Wg Cdr (Lt Col) Muhammad Shahid Gohar, MBBS, FCPS (ENT), Pakistan Aeronautical Complex Hospital, Kamra (Disstt: Attock), Punjab, Pakistan.

**Keywords:** Antifungal, Otomycosis

## Abstract

***Objectives*** *: *The aim of this study was to determine the frequency of otomycosis, the clinical presentation, predisposing factors and treatment outcomes.

***Methods:*** This observational study was conducted at ENT department of Combined Military Hospital Attock, from October, 2010 to September, 2012. Convenient sample comprising 180 patients of both sexes and all age groups were selected from ENT OPD. The frequency, predisposing factors and most common symptoms of otomycosis were recorded. The response to different antifungal agents was also observed. Results were recorded in percentages.

***Results: ***There were 180 patients with documented diagnosis of otomycosis. There were 107 (59%) males and 73 (41%) females. The age of patients ranged from 1½ years to 75 years with a mean age of 38.5 years. Mean follow up time was 2 years. Most common presenting symptom was hearing loss (77.7%) followed by pruritis (68.8%) and otalgia (40%). We prescribed 1% clotrimazole drops or lotion in 58% patients and 2% salicylic acid in 31% cases. Both of these agents are effective. Topical 1% clotrimazole drops yielded highest resolution rate with lowest recurrent rate. Overall 149 (83%) patients were improved with initial treatment and 31 (17%) did not respond to initial treatment. Eight (4.4%) patients had a history of otological procedures. Four (2.2%) patients had canal wall down procedures that resulted in mastoid cavity. To analyse the efficacy of 1% clotrimazole and 2% salicylic acid we applied Z-Test to calculate the difference between 2 proportions of patients before treatment with those patients who remained uncured after treatment.

***Conclusion: ***Otomycosisis commonly presented with decreased hearing, pruritis, otalgia & otorrhoea. It usually resolves with local toilet of ear and instillation of antifungal agents. Eradication of disease is difficult in presence of a mastoid cavity and metabolic diseases like diabetes mellitus.

## INTRODUCTION

Otomycosis Is a common condition encountered in a general otolaryngology clinic setting and its prevalence in this study is 7% among patients who presented with signs and symptoms of otitis externa. It is almost in accordance with other studies.^1^ It is a pathologic entity, with candida and aspergillus the most common fungal species.^2^^.^^3^ It is not clear that the fungi are the true infective agents or mere colonization species as a result of compromised local host immunity secondary to bacterial infection. Various predisposing factors include a humid climate, presence of cerumen, instrumentation of the ear, increased use of topical antibiotics / steroid preparations^4^, immunocompromised host, patients who have undergone open cavity mastoidectomy and those who wear hearing aids with occlusive ear mold. The infection is usually unilateral and characterized by inflammatory pruritis, scaling and otalgia.^5^ Treatment recommendations have included local debridement, local and systemic antifungal agents and discontinuation of topical antibiotics. Sometimes otomycosis presents as a challenging disease for its long term treatment and follow up, yet its recurrence rate remains high.^6^

We conducted this study with the aim to determine the frequency, common presenting symptoms, predisposing factors and outcome of different treatment modalities.

## METHODS

It was observational study conducted over two years from 2010 to 2012 in ENT Department of Combined Military Hospital Attock. Patients were prospectively recruited via non-probability convenience sampling. It composed of 180 patients of both sexes and all age groups with documented diagnosis of otomycosis.

Data was collected regarding presenting frequency of the disease, response to different treatment regimens, common symptoms, history of prior otological procedures, treatment outcomes and follow up duration.

Statistical analysis was carried out. The diagnosis of otomycosis was made on the basis of the recognizable and characteristic appearance of fungal debris and fruiting bodies under microscopy. Cultures are not routinely obtained because there is generally a rapid response to treatment in most of the cases. The treatments offered to most of patients are as follow. Clotrimazole 1% lotion or cream was used after cleaning the canal with the use of microscopy and gauze impregnated in clotrimazole cream. Most of the cases settled in one week. The treatment was continued for three weeks in resistant cases. Prior treatments included ototopical or oral preparations received before presentation. Successful treatment outcome was defined as resolution of all evidence of fungal infection on physical examination. Residual disease was defined as a condition that failed to respond to our initial choice of treatment. Recurrent disease was defined as a condition that occurred in patients who had resolution of disease after initial treatment but recurred in the same ear at a later date.

## RESULTS

A total of 180 patients with documented diagnosis of otomycosis were included in the analysis. The group consisted of 107 (59%) males 73 (41%) females. The age at diagnosis ranged from 1½ to 75 years with a mean age of 38.25 years and a median age of 30 years. Mean follow up time was 2 years, bilateral disease was observed in 36 (20%) patients on initial presentation. The presenting complaints at the time of diagnosis are tabulated in Table-I.

**Table–I:** Symptoms at the time of diagnosis

*S. No.*		*No. of patients*	*%*
1.	Hearing loss	140	77.7
2.	Pruritis	124	68.8
3.	Otalgia	72	40
4.	Otorrhoea	57	32
5.	Tinnitus	10	5.5

As shown, hearing loss and pruritis were the most common symptoms at the time of diagnosis, followed by otalgia, otorrhoea and tinnitus. Physical examination findings that suggest otomycosis include a thick fibrinous accumulation of debris, small well circumscribed areas of granulation tissue within the external canal or on the tympanic membrane and watery discharge. Treatment received before diagnosis is listed in Table-II.

**Table–II:** Therapeutic Agent

*S. No.*		*No. of patients*	*%*
1.	Ototopical antibiotic drops	110	61
2.	Pain killer	132	73
3.	Antihistamine	115	64
4.	Systemic antibiotic	90	50
5.	Soda Glycerin E/D (others)	80	44

The duration of treatment ranged from days to years. Nearly 60% patients have been using ototopical antibiotic drops, neomycin, polymyxin-B hydrocortisone and ciprofloxacin and oral antimicrobial for treatment of presumed otitis media before diagnosis.

Disease complications included serous otitis media in 57 patients (30%), TM perforation in 28 patients (15%) and external auditory canal osteitis in 9 patients (5%). Tympanic membrane perforations were considered a complication of otomycosis if they were present during initial presentation and healed with resolution of infection or if they were observed to occur during the course of treatment. Of the 9 patients with osteitis, 4 had a known history of diabetes. Among all subjects, diabetes was a documented co morbidity in 9 (5%) patients, though this is not significantly different from the reported prevalence of diabetes in the general population.^7^ The most common therapeutic options used in our practice are listed in Table-III.

**Table–III:** Treatment agent

*S. No.*		*No. of patients*	*%*
1.	Clotrimazole lotion or cream 1%	105	58
2.	2% salicylic acid in alcohol	55	31
3.	Ototopical drops with propylene glycol	20	11
4.	Antimicrobial	45	25
5.	Fluconazole	8	4.4
6.	Nilstat oral drops	40	22

The therapeutic agents were always used in conjunction with thorough mechanical debridement of visible fungal elements in the external auditory canal. 1% clotrimazole and 2% salicylic acid appeared to be effective but 1% clotrimazole is slightly more effective. Treatment duration ranged from 1-8 weeks. Over all 149 (83%) patients improved with initial treatment. Twenty seven (16%) patients were lost to follow up after initiation of treatment, and 31 (17%) failed initial treatment.

Among the 149 patients that responded to initial treatment 68 (38%) patients had recurrent disease. The efficacies of the two most common treatment modalities are shown in the Table-IV.

**Table–IV:** Efficacy of different treatment modalities

	*1% Clotrimazole*		*2% Salicylic Acid*	
	*No. of patients - 105*	*%*	*No. of patients - 55*	*%*
Resolution	85	81	47	85
Residual disease	20	19	8	15
Recurrent disease	50	48	14	25

To analyze the efficiency of 2% salicylic acid and 1% clotrimazole, we applied Z-Test to calculate the difference between 2 proportions of patients suffering from symptoms of disease before starting treatment with those patients who remained uncured after completing the treatment. The Z-values were calculated which were away from critical region. We draw the conclusion that the difference between two proportions is significant. We tested the two treatment modalities and the result of statistical tests show that both treatment regimens are effective but 2% clotrimazole is slightly more effective.

There are 4 cases of documented external auditory canal dermatitis as a result of local irritation from clotrimazole otic solution. Culture results were available for 10 patients, two of aspergillus niger and five of aspergillus flavors and three for candida species. Eight (4.4%) patients had history of otologic procedure in the affected ear that ranged from tympanostomy tube placement to tympano mastoidectomy. Four (2.2%) patients had canal wall down procedures that resulted in a mastoid cavity. These patients were treated with topical 1% clotrimazole lotion. Residual fungal disease after initial treatment was seen in one patient and one had recurrent disease.

## DISCUSSION

Otomycosis is a superficial mycotic infection of the outer ear canal frequently encountered by otolaryngologist and can usually be diagnosed by clinical examination. However the correct diagnosis requires a high index of suspicion. The infection may be either sub acute or acute and is characterized by inflammation, pruritis, scaling and severe discomfort. The mycosis results in inflammation, superficial epithelial masses of debris containing hyphae, suppuration and pain. In addition, symptoms of hearing loss and aural fullness are as a result of accumulation of fungal debris in the canal. Pruritis has been frequently cited as one of the hallmark symptoms up to 93% in one study.^8^^.^^9^ It was reported among the chief complaints in 108 (77%) of the current study population. Aspergillus and Candida species are the most commonly identified fungal pathogens in otomycosis.^10^^.^^11^ Infections with Candida can be more difficult to detect clinically because of its lack of a characteristic appearance like aspergillus and can present as otorrhoea not responding to aural antimicrobial.^12^ Otomycosis attributed to Candida is often identified by culture data. Although multiple in vitro studies have examined the efficacy of various antifungal agents, there is no consensus on the most effective agent.^13^ Various agents have also been used clinically with variable rate of success.^14^^.^^15^ Nevertheless, application of appropriate topical antifungal agents coupled with frequent mechanical debridement usually results in prompt resolution of symptoms, although recurrent or residual disease can be common. In this series more than 70% of the patients had resolution of the infection with initial treatment, often in less than two weeks. Topical clotrimazole is our preferred antifungal agents for its efficacy against both aspergillus and Candida species. There were only 4 (2.2%) cases of local sensitivity to clotrimazole and the infections seem to resolve faster and display a lower recurrence rate.

TM perforation and serous otitis media are not uncommon with otomycosis and tend to resolve with treatment. The pathophysiology of the TM perforation may be attributed to avascular necrosis of the TM as a result of mycotic thrombosis in the adjacent blood vessels. The rate of 20% (8 patients) of TM perforation in this series is similar to that observed by Pradhan et al.^9^ There were no clinical features predictive of TM perforation. TM involvement is likely a consequence of fungal inoculation in most medial aspects of the external canal or direct extension of the disease from adjacent skin.

There appear to be little consensus with respect to the predisposing factors for otomycosis. For instance the presence of cerumen has been speculated to be supportive of fungal growth by some, yet inhibitory by others.^1^^,^^8^^,^^15^ There have also been reports of autoinoculation of ear canal that result in otomycosis by patient with untreated dermatomycosis. More recently there has been increasing concern with respect to increasing incidence of otomycosis from wide spread use of fluoroquinolone otic drops.^16^ In this series neomycin-polymyxin Bnoted in 8% of the patients in the study appears to increase the risk for developing otomycosis. This is higher than that reported in other series such as that by Pradhan et al,^9^ where 4.6% of the subjects were post mastoidectomy patients. The data appear to support prior otologic procedures particularly that result in a mastoid cavity, as a potential risk factor for otomycosis. Several factors may contribute to the development of otomycosis in the previously operated ear. First recurrent drainage or subsequent antibiotic / antiseptic application may alter the local environment of the external canal and allow super infection of nosocomial fungi. Second alteration of the anatomy by canal wall down procedures may also produce changes in cerumen production or relative humidity that favor fungal growth. This suggests that eradication of disease is more difficult in the presence of mastoid cavity. The addition of oral antifungals is reserved for cases with severe disease and poor response to topical therapy. We believe oral antifungals are unlikely to succeed in the absence of adequate local care. The limitation of this study is the increase heat and humidity in our geographical region which may limit the applicability of these findings in regions with a more temperate climate. Future research may include better characterization of the effective treatment dose and duration of the various available antifungal agents.

## Conclusions

This study demonstrates that the diagnosis of otomycosis requires vigilance from clinicians given its non specific symptoms. Treatment regimens such as clotrimazole and 2% salicylic acid coupled with mechanical debridement are generally effective. However recurrence is not uncommon and eradication of disease can be particularly difficult in post mastoidectomy patients and in immunocompromised patients.
